# Thermally Modified Palygorskite Usage as Adsorbent in Fixed-Bed Reactor for High Concentration NH_4_^+^-N Removal and Further Application as N—Fertilizer in Hydroponic Cultivation

**DOI:** 10.3390/ma15196541

**Published:** 2022-09-21

**Authors:** Christina Vasiliki Lazaratou, Stylianos Dimitrios Panagopoulos, Dimitrios V. Vayenas, Dionisios Panagiotaras, Dimitrios Papoulis

**Affiliations:** 1 Department of Geology, University of Patras, GR-26504 Patras, Greece; 2 Department of Chemical Engineering, University of Patras, GR-26504 Patras, Greece; 3 Institute of Chemical Engineering Sciences, Foundation for Research and Technology, GR-26504 Patras, Greece; 4 Department of Environment, Ionian University, M. Minotou-Giannopoulou 26, GR-29100 Zakynthos, Greece

**Keywords:** clay mineral, ammonium, adsorption, kinetic models, N-fertilizers

## Abstract

Palygorskite sample (Pal) underwent thermal treatment at 400 °C (T-Pal) to be used as adsorbent for the removal of 200 mg NH_4_^+^-N/L from artificial solution. After thermal treatment, the sample was characterized via X-ray diffraction (XRD) and scanning electron microscopy (SEM). For NH_4_^+^-N removal, T-Pal was added as a bed matrix in fixed-bed reactor experiments and the effect of flow rate was determined. It was indicated that with the flow rate increase from 10 mL/min to 50 mL/min, fewer liters of the solution were purified, rendering a longer residual time of interactions, which is optimal for NH_4_^+^-N removal from T-Pal. The maximum removed amount was calculated at 978 mg NH_4_^+^-N (*q_total_*), suggesting T-Pal is a promising ammonium adsorbent. The data of kinetic experiments were applied to Clark, Yoon–Nelson, and Thomas kinetic models, with Clark having the best fit, highlighting a heterogenous adsorption. At the end of kinetic experiments, T-Pal applied in hydroponic cultivations and presented a sufficient release rate, which was found utilizable for saturated T-Pal usage as N fertilizer that satisfactory results were deemed concerning lettuces characteristics and growth.

## 1. Introduction

Palygorskite (Pal) belongs to the fibrous clay minerals with a typical 2:1 ribbon-like structure [1]. In parallel with these SiO_4_ ribbons, channels are extended at an approximate width of 7.3 Å [2] which are full of exchangeable cations, coordinated and zeolitic water molecules that balance the layer charge [1]. The specific structural arrangement is responsible for Pal porosity, high specific surface area (SSA > 200 m^2^/g), and sorption capacity [3]. At temperatures above 300 °C, the zeolitic water is lost, whereas above 350 °C part of coordinated water is lost as well, and dehydroxylation starts taking place. Water molecules’ absence and dehydroxylation lead to emptying space of the channels, as well as the reveal of more active sites (-OH groups), respectively, allowing the adsorption of more, or even bigger size cations [2,4]. Based on these enhanced-adsorption properties, thermally modified palygorskite (T-Pal) was previously used for a variety of contaminant removal from water systems, such as dyes [5,6,7], heavy metals [8], or radioactive uranium [9].

Moreover, T-Pal was previously applied as a low-concentration ammonium adsorbent in water systems, achieving removal efficiencies exceeding 75% [10,11]. Ammonium (NH_4_^+^) can be a common inorganic contaminant in groundwater, mostly derived from N-fertilizer consumption and animals’ feedlots, but is also the most frequent nitrogenous pollutant in wastewaters at varying concentrations (10–1000 mg L^−1^) [12]. Municipal wastewater, landfill leachates, livestock, poultry, and industrial wastewaters are the main sources of water bodies contamination from NH_4_^+^/NH_3_, which can finally lead to eutrophication and toxicity to humans and other organisms [13]. A variety of methods has been examined for the immense NH_4_^+^ removal from the wastewaters, among them air stripping [14], membranes [15], or adsorption [16,17], whereas from these techniques, the NH_4_^+^ could be recovered [13].

The recovery of NH_4_^+^ can be essential since N is a crucial element for agriculture and plant growth which can be provided by ammonium solely or in combination with nitrate [18,19]. A great advantage of the adsorption method is the usage of low-cost, environmentally friendly, and in many cases natural materials, such as Pal or zeolite as adsorbents. Natural materials, saturated with NH_4_^+^ or other nutrients can potentially be further exploited without causing secondary pollution, such as fertilizers in cultivations, even in hydroponics [16,20,21].

The present study highlights the potential application of thermally treated palygorskite (T-Pal) as an adsorbent of high concentration NH_4_^+^-N that could be representative of wastewaters. T-Pal was applied as the support media on a lab-scale fixed-bed reactor, for the conduction of continuous flow rate experiments till saturated. The N-saturated T-Pal was further used in hydroponic cultivations for lettuce growth for N-supply, as an environmentally friendly and cost-effective solution for N recovery. The kinetics of the fixed-bed reactor experiments were fitted in Clark, Yoon–Nelson, and Thomas’s kinetic models, while the evaluation of lettuce characteristics was analyzed via ANOVA statistical analysis.

## 2. Materials and Methods

### 2.1. Sample Preparation and Characterization

Pal sample was provided by Geohellas S.A. (Grevena, Greece) at specific grain size of 1.4–2.36 mm, which was washed and dried at 50 °C. The dried sample was further thermally treated at 400 °C in a controlled muffled oven for 2 h and then cooled at room temperature in a desiccator. The temperature of 400 °C was selected since it is the minimum where both zeolitic water loss and dehydroxylation may take place within the sample, thus, ensuring structural changes and cost-effectiveness [5]. The X-ray fluorescence (XRF) measurements of the major (SiO_2_, Al_2_O_3_, CaO, MgO, MnO, Fe_2_O_3_, K_2_O, Na_2_O, P_2_O_5_, TiO_2_) elements were performed. An amount of 1.8 g of dried ground sample was mixed with 0.2 g of wax (acting as a binder) and pressed on a base of boric acid to a circular powder pellet of 3.2 cm in diameter. Analyses were performed with a RIGAKU ZSX PRIMUS II spectrometer, which was equipped with an Rh anode running at 4 kW, for major and trace element analysis. The spectrometer was equipped with the diffracting crystals: LIF (200), LIF (220), PET, Ge, RX-25, RX-61, RX-40, and RX-75. X-ray diffraction (XRD) patterns were obtained for the samples in a 2θ range of 2° to 60° and at a scanning rate of 2 °/min, using an XRD Bruker D8 advance diffractometer, with Ni-filtered CuKα radiation (λ = 1.5418 Ǻ). Their typical morphological characteristics were verified with scanning electron microscopy (SEM), using an SEM LEO SUPRA 35VP. Fourier transform infrared spectroscopy (FTIR) spectra were obtained using FTIR spectrophotometer Spectrum (Patras, Greece) RXI (Perkin Elmer) at room temperature. The samples were prepared by mixing 0.1 mg of Pal, T-Pal, and T-Pal after NH_4_^+^-N adsorption with KBr, and then were pressed till pellets were formed. The wavenumbers range from 400 cm^−1^ to 4000 cm^−1^ and were analyzed using Spectrum v5.3.1 software. The Cations Exchange Capacity (CEC) was determined using sodium acetate, according to the US-EPA 9081 method.

### 2.2. Fixed-Bed Reactor Experiments

**Figure 1:**
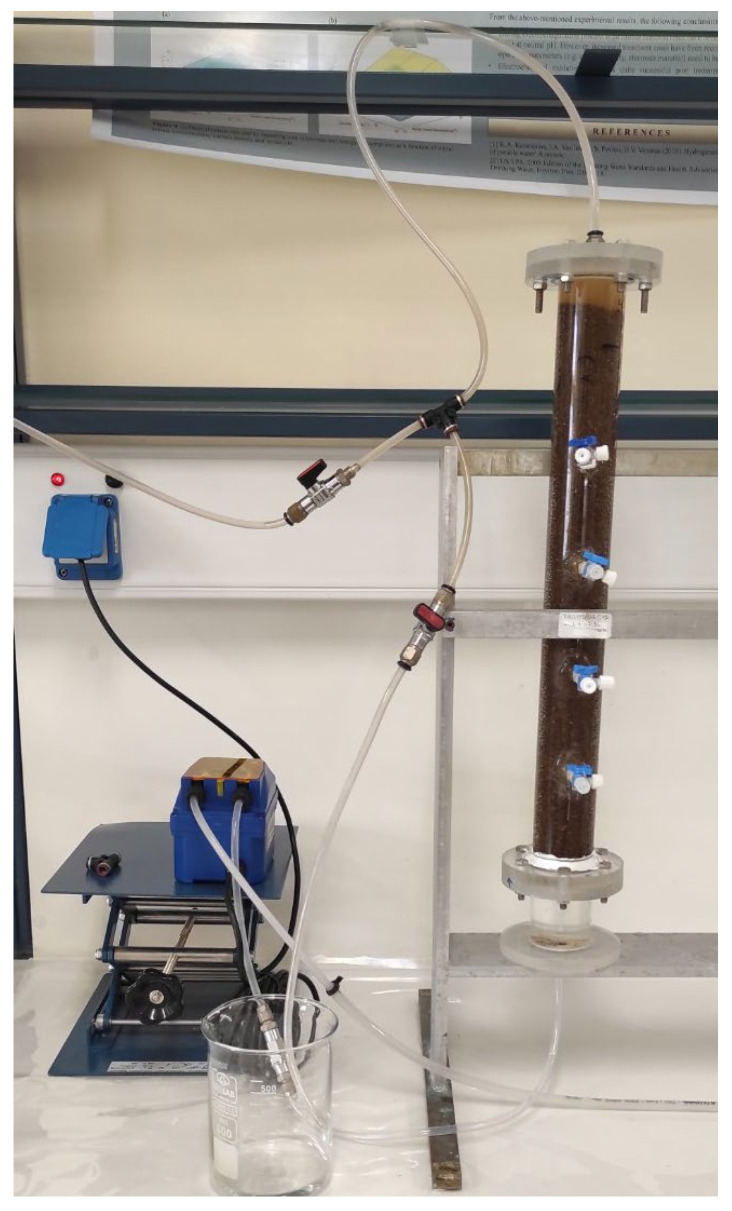
The Plexiglas column used for the experimental setup.

The adsorption experiments on fixed—bed reactors were conducted on Plexiglas tubes (columns) of laboratory scale (Figure 1). The tube dimensions were 40.0 cm in height and 4.0 cm internal diameter, with 450 mL operational volume, equipped with four sampling valves at various heights. The column was firstly filled with 288 g of T-Pal sample at 1.4–2.36 mm grain size and 0.67 gr cm^−3^ density. The removal of NH_4_^+^-N was examined using NH_4_Cl standard solution of 200 mg/L initial concentration which was supplied after the column was filled with the matrix. The specific concentration was selected to be comparable to previous experiments with zeolite from Kotoulas et al. [16], which were conducted at the same laboratory and equipment. The effect of continuous flow rate (15, 35, and 50 mL/min) was considered. Various samples were selected at various time intervals till the saturation of T-Pal was achieved and the breakthrough curve could be formed. Each sample was centrifuged at 5.500 rpm for 3 min and filtrated through Whatman filters (0.45 μm) to remove the finest suspended.

The final concentration of each time interval was measured at UV–Vis spectrophotometer at 625 nm according to modified Salicylate method of Verdouw et al. [22]. From the data obtained, the adsorption capacity *(q_e_*) and maximum removal efficiency (Y%) were determined based on Equations (1)–(4).

(1) $$q_{total}=\frac{Q}{1000}\int_{t=0}^{t=t_{total}}C_{ads}dt$$

(2) $$q_{e\left(\exp\right)}=\frac{q_{total}}{x}$$

(3) $$W_{total}=\frac{C_{0}Qt_{total}}{1000}$$

(4) $$Y=\frac{q_{total}}{W_{total}}\times 100$$

where *q_total_* is the amount of adsorbed NH_4_^+^-N (mg) and was calculated with the area method [23], *Q* is the applied flow rate (mL/min), *t_total_* is the 90% of time needed for adsorbent saturation (min), *C_ads_* the adsorbed NH_4_^+^-N amount (mg^/^L) (*C_ads_ = C_0_ − C*), *x* the total mass of the adsorbent and *W_total_* the total amount of NH_4_^+^-N (mg) sent to the column.

### 2.3. Kinetic Models

Some of the most representative models to express the breakthrough curve results are the Thomas, Yoon–Nelson, and Clark kinetic models. These models can adequately determine the maximum solid-phase concentration, which can be estimated at 50% of bed saturation approximately [24].

Specifically, the Thomas model expresses the second-order reversible reaction kinetics, while assuming that adsorption follows the Langmuir isotherm. The linearized Thomas equation is described in Equation (5):

(5) $$ln\frac{C_{0}}{C_{t}}=\frac{K_{Th}q_{max}m}{F}-K_{Th}C_{0}t$$

where *C*_0_ the initial pollutant concentration (mg/L), *C_t_* is the pollutant concentration in the effluent (mg/L), *F* the flow rate (ml/min), *K_Th_* is the Thomas adsorption rate constant (L/mg min); *m* the adsorbent mass (g) and *q_max_* is the maximum adsorption capacity (mg/g).

The Yoon–Nelson model is the simplest of the fixed-bed studies since it does not include the bed parameters or detailed data about the adsorbent-adsorbate interactions [25]. It is a model based on the decrease in the adsorption probability, and the most concise to predict the concentration change till the adsorbent breakthrough. The linearized Yoon–Nelson model is expressed as presented in Equation (6):

(6) $$ln\frac{C_{t}}{C_{0}-C_{t}}=K_{YN}t-K_{YN}t_{50}\;$$

where *C*_0_ the initial pollutant concentration (mg/L), *C_t_* is the pollutant concentration in the effluent (mg/L), *K_YN_* the Yoon–Nelson constant (1/min), *t* time (min) and *t*_50_ the required time for adsorbate breakthrough (min).

The Clark model is based on the adsorption equilibrium and mass transfer phenomenon to predict the breakthrough curve, while also assumes that the Freundlich isotherm expresses the adsorbent–adsorbate interactions [25]. The linearized Clark model is expressed as in Equation (7).

(7) $$ln[{\left(C_{t}/C_{0}\right)}^{n-1}-1]=-r^{\prime }t+lnA$$

where *C*_0_ the initial pollutant concentration (mg/L), *C_t_* is the pollutant concentration in the effluent (mg/L), *n* the Freundlich constant derived from the batch tests, *r* and *A* the Clark constants (1/min) and *t* time (min).

### 2.4. Determination of Error Functions and Coefficients

The parameters of each nonlinear kinetic model are the result of linearizing the non-linear model expressions and least squares fit [26]. For comparison and quantification of the deviation and to determine the uncertainty in error distribution, the coefficient of determination (*R*^2^) and the sum of squares of errors (SSE) were determined for both linear and nonlinear models as it is expressed in Equations (8)–(10), respectively.

(8) $$R^{2}=\frac{\sum_{i=1}^{p}{\left(q_{e,theor}^{i}-{\bar{q}}_{e,means}^{i}\right)}^{2}}{\sum_{i=1}^{p}[(q_{e,theor}^{i}-{\bar{q}}_{e,means}^{i})+\left(q_{e,theor}^{i}-q_{e,means}^{i}{)}^{2}\right]}$$

(9) $$\mathrm{SSE}=\sum_{i=1}^{p}{\left(q_{e,theor}^{i}-q_{e,means}^{i}\right)}^{2}$$

(10) RMSE=1n−1 ∑i=1p(qe,theori−qe,meansi)2

where *i* is the experimental run, *q_e,theor_* is the theoretically evaluated equilibrium values from the model (mg/g) and *q_e,means_* (mg/g) is the experimental equilibrium data (mg/g).

### 2.5. Hydroponic Cultivations

The impact of NH_4_^+^-saturated samples on hydroponic lettuce (*Lactuca sativa* L.) cultivations as nitrogen fertilizers in laboratory-scale, was estimated based on the study of Urlić et al. [19], where various ammonium concentrations were examined as the sole N-supply source. Tap water from University of Patras was used as the nutrient solution as it contained 5 mg NO_3_^−^-N/L, 0.02 mg NH_4_^+^-N^/^L, and other nutrient minerals, mostly Ca^2+^ and Mg^2+^ as can be seen in Table 1. There was no synthetic nutrient solution since the study is focused on the interactions of T-Pal/NH_4_^+^-N release and NH_4_^+^-N^/^roots uptake. The added mass of T-Pal was 15.8 ± 0.32 g/L in tap water in order to release 3 mmol/L NH_4_^+^ as N—fertilizer, according to the optimal conditions that were determined in the study [19]. The exact mass was determined based on the T-Pal adsorption capacity from the fixed-bed reactor experiments. Additionally, there was a control system where lettuce cultivations were growing in tap water only. The cultivation consisted of seedlings of 5 cm height in 51 mm diameter hydroponic pots, filled with inactive expanded clay for plant support. The total volume capacity of the used hydroponic boxes was 1 L per seedling. The NH_4_^+^-N and NO_3_^−^-N consumption was measured three times per week. pH was not adjusted and remained neutral at 7.1 ± 0.3, while room temperature was stable at 20 ± 1 °C. On day 40 of the experiment, the final height, shoot, root lengths, and wet shoot and root weights were measured after the lettuce plants harvest. The shoots and roots were further dried at 45 °C for 48 h and then measured again for dry shoot and root weights. Statistical analysis was performed between hydroponic systems using single-factor analysis of variance (ANOVA). The statistical significance of *p* < 0.05 was set using the one-way ANOVA program in Microsoft Excel 2010.

## 3. Results and Discussion

### 3.1. Characterization of Pal before and after Thermal Treatment

**Figure 2:**
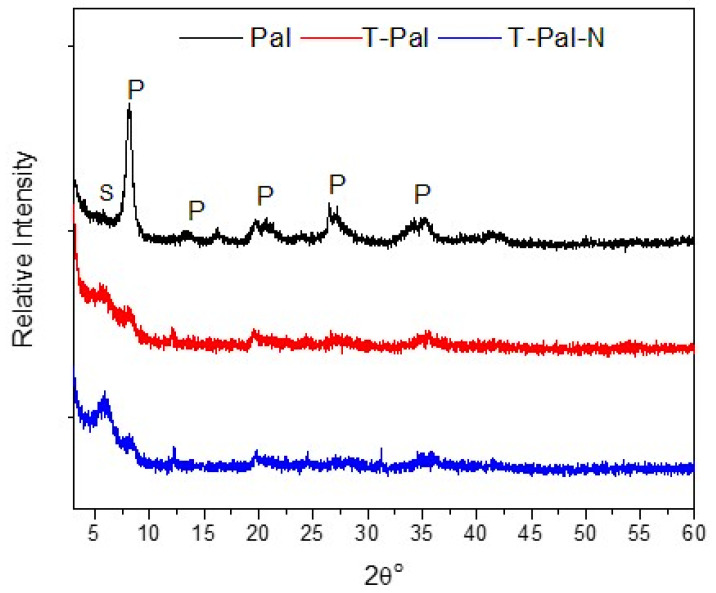
XRD patterns of raw Pal and thermally treated T-Pal samples, and T-Pal after NH_4_^+^-N adsorption (T-Pal-N). (S: saponite, P: palygorskite).

The chemical composition information was provided with X-ray fluorescence (XRF). It was indicated that Pal composition was 63.7% SiO_2,_ 1.10% Al_2_O_3_, 19.8% MgO, 9.4% Fe_2_O_3_, 0.3% CaO, 0.4% MnO, 0–0.06 of N_2_O, KOH, TiO_2_, and P_2_O_5_ and 10% LOI. Additionally, the CEC of palygorskite was estimated at 30 meq/100 g. The mineralogical composition of the palygorskite sample before and after thermal treatment was identified via the X-ray diffraction (XRD) method, whereas T-Pal after NH_4_^+^-N was also analyzed for potential alterations after adsorption. As shown in Figure 2, palygorskite is the dominant mineralogical phase of the sample, while saponite impurity was also detected, since these two clay minerals co-exist in Ventzia Basin (Grevena, Greece) [27]. The typical palygorskite reflections can be observed at 2*θ*^ο^ values 8.3°, 20°, 27°, and 34°, while the reflection at 6° is referred to as saponite. After thermal treatment, differences were shown in T-Pal reflections, since the intensity of the first characteristic reflection at 8.3° was decreased, contrary to the saponite reflection which showed sharper. In the study of Chen et al. [5], decreased palygorskite reflections were recorded, while in the present study, new reflection at 30° was formed, most likely by dehydration and structural rearrangement [28]. After the adsorption of NH_4_^+^-N on T-Pal at the fixed-bed reactor (T-Pal-N), there are no significant differences, showing that the T-Pal structure was unaffected after the interaction with NH_4_^+^ -N. More specifically, the most notable alteration at the spectra was a slight peak shifting at the basal reflections of 20° (Figure 1), while the reflections were slightly decreased. The intensity change in the reflections renders the surficial interaction between adsorbent–adsorbate most probable [29], whereas the peak shifting can be attributed either to the ion exchange that took place or to the surficial bonding that may affect the crystallinity. Concerning the reflection of saponite, at T-Pal-N sample was steeper than palygorskite, displaying potential surficial H-bonded interactions [30].

**Figure 3:**
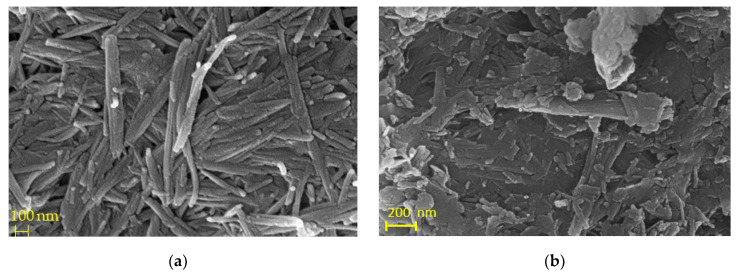
Scanning electron microscopy (SEM) images from (**a**) Pal sample at 100 nm and (**b**) T-Pal sample at 200 nm.

Furthermore, the fibrous morphology was verified with scanning electron microscopy (SEM). The typical fibers are depicted in Figure 3, showing fibers with lengths ranging from 250 nm to 1 μm. The effect of thermal treatment at 400 °C was significant (Figure 3a). Specifically, T-Pal fibers (Figure 3b) were strongly agglomerated (bundles) while their length decreased as a result of the total loss of coordinated water molecules [31].

**Figure 4:**
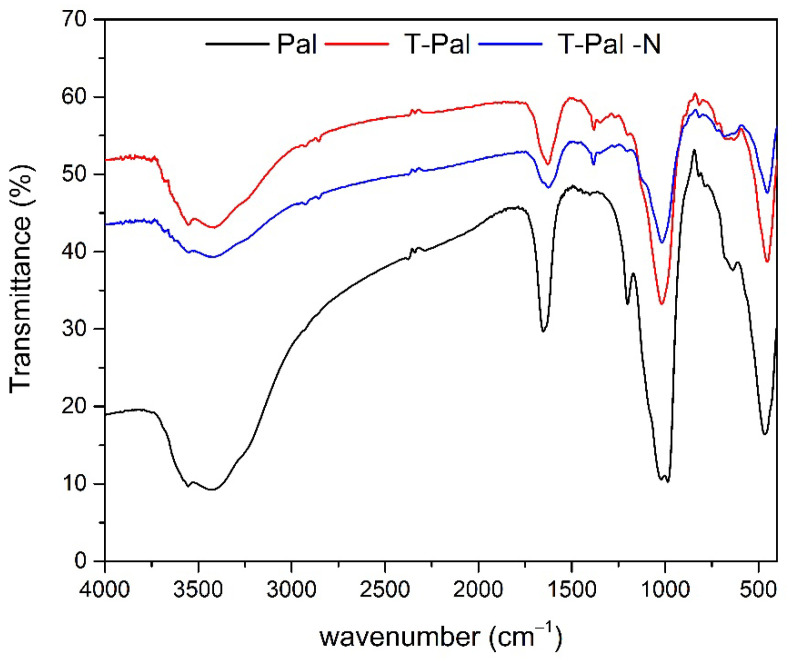
FTIR spectra of raw Pal and thermally treated T-Pal samples, and T-Pal after NH_4_^+^-N adsorption (T-Pal-N).

Concerning the FTIR analysis, the spectra of Pal, T-Pal, and T-Pal-N can be shown in Figure 4. At 478 cm^−1^, 1030 cm^−1^, and 1120 cm^−1^, typical bands of Si-O stretching were presented, whereas the bands at 985 cm^−1^, 1024 cm^−1^, and 1120 cm^−1^ are typical of the palygorskite [28], and the band of 1200 cm^−1^ is characteristic of the ribbon structure [32]. After thermal treatment, the mineralogical phase was not influenced; however, most of the bands presented decreased vibration, and a slight shifting at ~3400 cm^−1^ to ~3600 cm^−1^ that was followed and can be attributed to a partial loss of bound water after the thermal treatment. Moreover, after NH_4_^+^-N adsorption on Pal (T-Pal-N), an intense band at 1380 cm^−1^ can be attributed to the newly formed N-H bond, rendering the ammonium removal on T-Pal takes place [33].

### 3.2. Fixed-Bed Reactor Experiments

#### 3.2.1. Effect of Flow Rate

**Figure 5:**
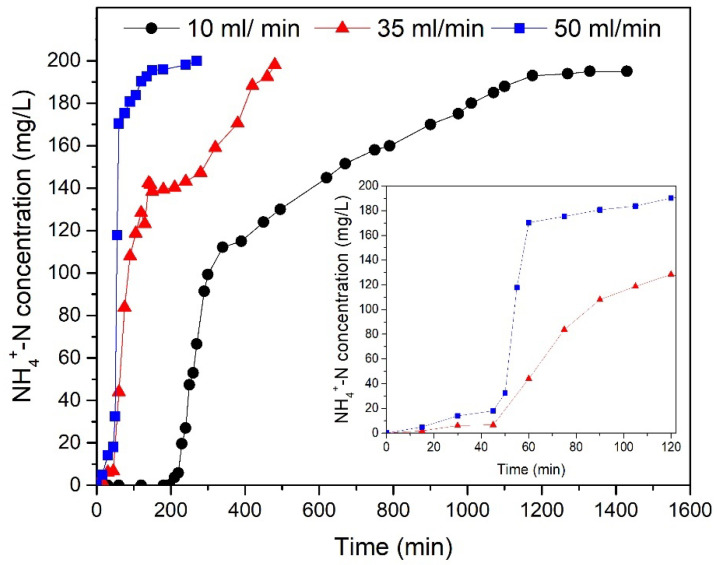
Effect of 10, 35 and 50 mL/min flow rates for NH_4_^+^-N removal (200 mg NH_4_^+^-N/L). The zoom area displays the NH_4_^+^-N removal within 120 min for flow rates 35 mL/min (red) and 50 mL/min (blue).

The effect of the three different flow rates (10 mL/min, 35 mL/min, and 50 mL/min) was examined via continuous flow rate experiments in a fixed-bed reactor. It was observed that increased flow rates (35 mL/min and 50 mL/min) came with decreased time for the breakthrough point, whereas at 10 mL/min more than the double time was necessary for the T-Pal saturation (Figure 5). Specifically, 2.2 L of the solution was purified within 1500 min that the breakthrough curve ended, for 10 mL/min influent. This removal corresponds to 4.38 bed volumes instead of 3.48 and 2.98 for 35 mL/min and 50 mL/min, respectively. The lower the flow rate is, the longer the residual time of ammonium to interact with T-Pal active sites and interlayer space, resulting in higher removal efficiencies [24]. Moreover, the breakthrough curves under all the examined flow rates presented similar shapes, as well as the same NH_4_^+^-N adsorption behavior, which can be characterized by the highest possible removal at the beginning, continuing with the steep increased NH_4_^+^-N concentration in the effluent, and ending at the almost stable final concentration where almost there is no adsorption. The first and final steps can be better depicted at a 10 mL/min flow rate, but the steep concentration increase can be clearly shown at a 50 mL/min flow rate (Figure 5).

At the end of the kinetic experiments, the operational parameters were determined. Table 2 shows the maximum NH_4_^+^-N removal efficiency (Y%) and adsorption capacity (*q_e_)* of T-Pal, as well as the liters of water that were treated during the 90% of saturation that was calculated based on Equations (1)–(4). The same equations were applied for time *t,* which represents the time needed to produce an effluent NH_4_^+^-N concentration below the permeable limits for wastewaters, according to Greek legislation [34]. According to the calculations presented in Table 2, can be observed that the highest examined flow rate 10 mL/min achieved the highest removal efficiency and capacity, whereas more liters of water were treated till the breakthrough. Moreover, as shown in Table 3, more liters of the solution were purified effluent at the flow rate of 10 mL/min, which can be characterized as the most efficient for NH_4_^+^-N treatment. Additionally, the highest adsorption capacity (*q_e_*), and removal efficiency were obtained for 10 mL/min for time *t*. The decreased flow rate values provided longer residence time for interactions between wastewater and bed material, rendering that probably the intraparticle mass transfer is dominant [35].

The results obtained were compared to the previous study of Kotoulas et al. [16], where zeolite was used at the same dimension reactor (for the removal of 200 mg NH_4_^+^-N/L as well at 8 mL/min flow rate), aiming to the competitive analysis of T-Pal as adsorbent for NH_4_^+^-N. In general, zeolite is considered the most widespread aluminosilicate adsorbent for ammonium [16,17,36,37]; nevertheless, T-Pal achieved removal efficiencies were quite promising and worth to be evaluated. It must be admitted that almost double the zeolite dose (500 g) presented higher adsorption capacity under similar experimental conditions [16], which would still be higher if the same dosage of T-Pal was used. Nevertheless, the values are quite comparable, rendering T-Pal a competitive adsorbent for NH_4_^+^-N.

#### 3.2.2. Application of Kinetic Models

The results obtained from the kinetic experiments for all the flow rate values were evaluated with the application of each breakthrough curve on different kinetic models such as Clark, Yoon–Nelson, and Thomas.

**Figure 6:**
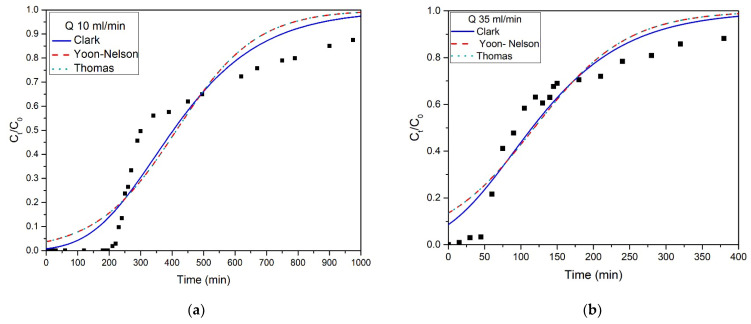

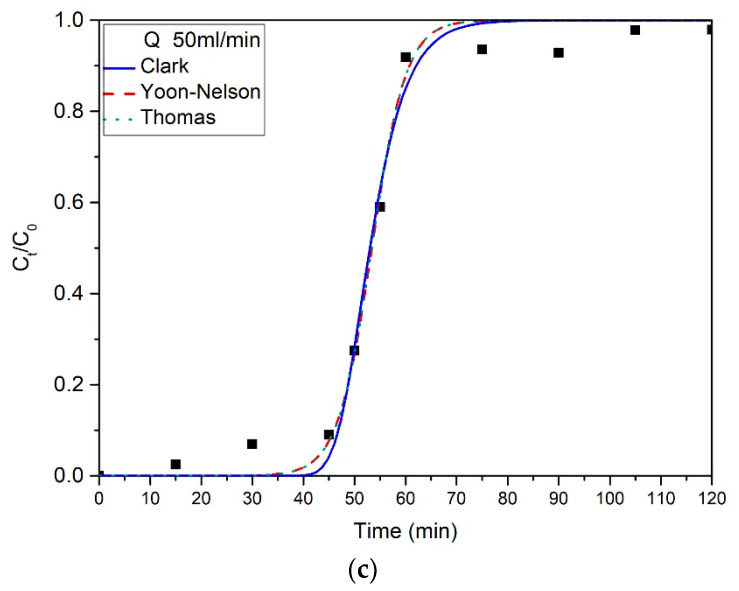
Fitting of Clark, Yoon–Nelson, and Thomas models for flow rate (**a**) 10 mL/min, (**b**) 35 mL/min, and (**c**) 50 mL/min.

In all cases, quite high values of correlation coefficient were obtained (R^2^ > 0.92), highlighting those models’ sufficiency to express the NH_4_^+^-N interactions with T-Pal. Table 4 displays the calculated parameters for the above-mentioned kinetic models for all the examined flow rates, whereas Figure 6 presents the exact fit of each model. Based on the R^2^ values, the Clark model noted the highest ones when 10 mL/min (0.942 > 0.929) and 35 mL/min (0.902 > 0.897) were the applied flow rates. Clark’s model associates the mass-transfer concepts and Freundlich isotherm [38]. The expression of kinetics via the Clark model indicates the heterogenous nature of adsorption, which also came in agreement with the data obtained from batch experiments conducted in the previous study [11], where Freundlich isotherm was most suitable to describe the removal of NH_4_^+^-N from T-Pal. However, with the flow rate increase at 50 mL/min, Thomas and Yoon–Nelson models yield slightly better R^2^ values than the Clark model (0.989 > 0.987), which can be attributed to the decreased mass transfer phenomena when the flow rate increases [38,39]. This can be verifying from the Thomas or Yoon–Nelson model values, in specific the K_th_ or K_YN_, which keep increasing with the flow rate increase as well, due to the lower mass transfer resistance, as was also reported in the study by Futalan and Wan [39].

The mass transfer phenomena found to have an impact on T-Pal and NH_4_^+^-N interactions, while as it was mentioned in Table 3, more liters of aqueous solution can be purified at lower flow rates, as well as it was shown from the difference in the optimal kinetic model fitting.

### 3.3. Hydroponic Cultivations for Lettuce Growth Using T-Pal for NH_4_^+^-N Supply

At the end of the kinetic experiments in the fixed-bed reactor, the used T-Pal was saturated with 978 mg of NH_4_^+^-N, as it was calculated based on Equation (1). As an N-enriched material, it was considered to be exploited as nitrogenous fertilizer in hydroponic cultivation of *Lactuca sattiva* L. The supply of 3 mmol of NH_4_^+^-N was found sufficient as lettuce nutrient [19], so it was selected to be examined in this case study as well, whereas 10% of NO_3_^−^-N would be also provided due to the tap water composition.

**Figure 7:**
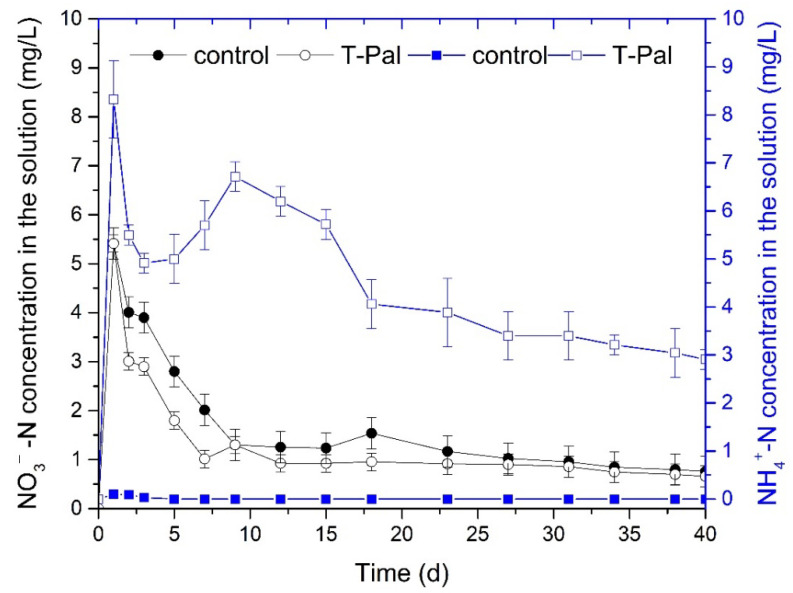
NH_4_^+^-N and NO_3_^−^-N concentrations over time in hydroponic solutions of control, and T-Pal-supplied systems for lettuce cultivation (pH: 7.1 ± 0.3, temperature: 21 °C).

**Figure 8:**
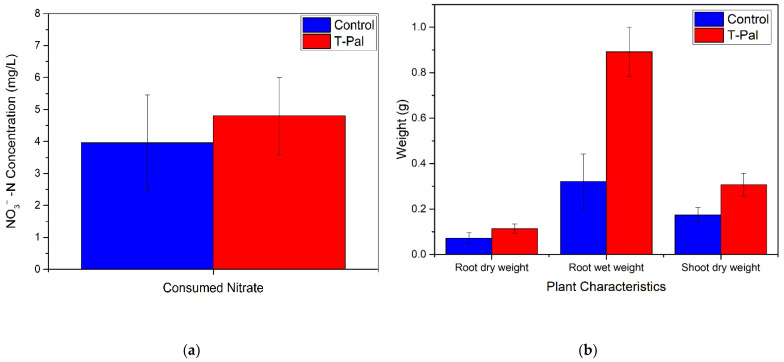
Parameters that presented statistical difference with ANOVA *p* values < 0.05 (**a**) NO_3_^−^-N uptake by lettuce plants (**b**) wet/dry root weight and shoot dry weight.

The overall 40-day experiments indicated that the added NH_4_^+^-N was not fully consumed from the lettuce plants (Figure 7), whereas the NH_4_^+^-N released amount from the saturated samples was less than it was expected, concerning the *q_total_*. This could be attributed to the strong interactions between NH_4_^+^ and clay minerals that continuous and intense agitation would be necessary to be broken. Despite that fact, the maximum ammonium was noted during the first day of hydroponics, and then steady consumption was observed (Figure 7). Moreover, it is important to mention that in the system with T-Pal, the NO_3_^−^-N consumption was higher compared to the control system, highlighting the boost in NO_3_^−^-N consumption under NH_4_^+^-N presence (Figure 7 and Figure 8).

Finally, the effect of N consumption on the plants’ characteristics was significant. Specifically, the produced lettuce plants provided with both NO_3_^−^-N and NH_4_^+^-N, gained much more shoot or root weight and length compared to the control system (Table 5). The enhanced N supply was further evaluated with the ANOVA statistical analysis, verifying the synergistic effect of NO_3_^−^-N/NH_4_^+^-N on plant growth. In detail, Figure 8a,b depict the quality characteristics of plants that presented statistical differences (*p* < 0.05). It can be observed the lettuces derived from the system with T-Pal presented enhanced root and shoot weight than the lettuces from the control system; however, the most crucial notification was the statistical difference at NO_3_^−^-N consumption. All the measurements were evaluated via statistical analysis during the 40-day cultivation, suggesting that the gradual consumption of nitrates was found to be optimal when ammonium co-exists, despite the fact that the final consumed concentration had no significant difference.

## 4. Conclusions

In the present study, experiments were conducted at fixed-bed reactors, using T-Pal of 1.4–2.3 mm as the support media for the removal of 200 mg NH_4_^+^-N from artificial solution. The effect of three different flow rates was examined, where in all cases, satisfactory removal rates were achieved, ranging from 33% up to 50% with a flow rate decrease from 50 mL/L to 10 mL/L. For the lower examined flow rate, more than 90% of the solution was purified, rendering 10 mL/min the most sufficient one. The data were fitted on kinetic models of Clark, Yoon–Nelson, and Thomas with increased distribution of A, K_YN_, and K_TH_ for each model, respectively, with the flow rate increase. The difference in these distribution yields the ammonium removal was affected by the mass transfer phenomena impact. The Clark model, which assumes that the Freundlich isotherm is dominant, was found to satisfactorily express the procedure, highlighting the heterogeneity of the interactions. Saturated T-Pal proved sufficient for N supply at the hydroponic cultivations, as the lettuces presented normal growth and better characteristics than the control system. The statistical analysis confirmed these results, especially about plant dry or wet weight. Further investigation for pilot scale application concerning NH_4_^+^ removal, or usage as fertilizers either in hydroponic cultivations or soils is worthy to be examined.

## Figures and Tables

**Table 1:** Physicochemical characteristics of University of Patras tap water.

Physicochemical Characteristics	Tap Water Sample
pH	7.3
Total Hardness (mg/L, CaCO_3_)	487
Ca^+2^ (mg/L)	159
Mg^+2^ (mg/L)	22
Na^+^ (mg/L)	26

**Table 2:** The NH_4_^+^-N removal efficiency (Y%), adsorption capacity (*q_e_*) of 287 g T-Pal, and liters of treated water (V_w_) till the end of breakthrough curve for 15, 35, and 50 mL/min flow rates.

Q (mL/min)	Y%	V_w_ (L)	*q_e_* (mg/g)
10	48.4	10.10	3.40
35	39.2	8.75	2.39
50	33.7	3.00	1.88

**Table 3:** The NH_4_^+^-N removal efficiency (Y%), adsorption capacity (*q_e_*) of 287 g T-Pal, and liters of treated water (V_w_) till the permeable nitrate limit for 15, 35, and 50 mL/min flow rates.

Q (mL/min)	Y%	V_w_ (L)	*q_e_* (mg/g)
10	99.8	2.20	1.53
35	95.8	1.75	1.17
50	92.7	1.50	0.97

**Table 4:** Kinetic parameters of nonlinear Clark, Yoon–Nelson, and Thomas kinetic models for NH_4_^+^-N adsorption from T-Pal.

Q (mL/min)	Clark					Yoon—Nelson					Thomas				
	*A*	*r*	*R* ^2^	*RMSE*	*SSE*	*K_YN_*	*τ*	*R* ^2^	*RMSE*	*SSE*	*K_TH_*	*q_TH_*	*R* ^2^	*RMSE*	*SSE*
10	0.931	0.005	0.942	0.093	0.295	0.078	413.59	0.929	0.109	0.407	3.94 × 10^−6^	2.88	0.929	0.109	0.407
35	3.472	0.017	0.902	0.101	0.195	0.193	114.69	0.897	0.108	0.225	9.65 × 10^−5^	2.79	0.897	0.108	0.225
50	7.824	0.212	0.987	0.162	0.023	0.299	53.58	0.989	0.045	0.018	1.50 × 10^−3^	1.86	0.989	0.045	0.018

**Table 5:** Shoot and root characteristics at the end of hydroponic cultivation.

Samples	Shoot Weight (g)	Shoot Dry Weight (g)	Shoot Height (cm)	Root Weight (g)	Root Dry Weight (g)	Root Height (cm)
T-Pal	2.630 ± 0.564	0.307 ± 0.005	13.30 ± 0.75	0.8919 ± 0.108	0.113 ± 0.020	22.5 ± 0.9036
Control	1.791 ± 0.065	0.174 ± 0.032	11.75 ± 0.25	0.3215 ± 0.220	0.072 ± 0.025	16.0 ± 1.0282

## Data Availability

Not applicable.
